# Enhancing fall risk assessment: instrumenting vision with deep learning during walks

**DOI:** 10.1186/s12984-024-01400-2

**Published:** 2024-06-22

**Authors:** Jason Moore, Robert Catena, Lisa Fournier, Pegah Jamali, Peter McMeekin, Samuel Stuart, Richard Walker, Thomas Salisbury, Alan Godfrey

**Affiliations:** 1 Department of Computer and Information Sciences, Northumbria University, Newcastle, NE1 8ST UK; 2 Department of Kinesiology and Educational Psychology, Washington State University, Pullman, USA; 3 Department of Nursing, Midwifery and Health, Northumbria University, Newcastle, UK; 4 Department of Sport, Exercise and Rehabilitation, Northumbria University, Newcastle, UK; 5 Northumbria Healthcare NHS Foundation Trust, North Tyneside, UK; 6 South Tyneside and Sunderland NHS Foundation Trust, Sunderland, UK

**Keywords:** Fall risk, Gait analysis, Object detection, Deep learning, Visual attention

## Abstract

**Background:**

Falls are common in a range of clinical cohorts, where routine risk assessment often comprises subjective visual observation only. Typically, observational assessment involves evaluation of an individual’s gait during scripted walking protocols within a lab to identify deficits that potentially increase fall risk, but subtle deficits may not be (readily) observable. Therefore, objective approaches (e.g., inertial measurement units, IMUs) are useful for quantifying high resolution gait characteristics, enabling more informed fall risk assessment by capturing subtle deficits. However, IMU-based gait instrumentation alone is limited, failing to consider participant behaviour and details within the environment (e.g., obstacles). Video-based eye-tracking glasses may provide additional insight to fall risk, clarifying how people traverse environments based on head and eye movements. Recording head and eye movements can provide insights into how the allocation of visual attention to environmental stimuli influences successful navigation around obstacles. Yet, manual review of video data to evaluate head and eye movements is time-consuming and subjective. An automated approach is needed but none currently exists. This paper proposes a deep learning-based object detection algorithm (*VARFA*) to instrument vision and video data during walks, complementing instrumented gait.

**Method:**

The approach automatically labels video data captured in a gait lab to assess visual attention and details of the environment. The proposed algorithm uses a YoloV8 model trained on with a novel lab-based dataset.

**Results:**

*VARFA* achieved excellent evaluation metrics (0.93 mAP50), identifying, and localizing static objects (e.g., obstacles in the walking path) with an average accuracy of 93%. Similarly, a U-NET based track/path segmentation model achieved good metrics (IoU 0.82), suggesting that the predicted tracks (i.e., walking paths) align closely with the actual track, with an overlap of 82%. Notably, both models achieved these metrics while processing at real-time speeds, demonstrating efficiency and effectiveness for pragmatic applications.

**Conclusion:**

The instrumented approach improves the efficiency and accuracy of fall risk assessment by evaluating the visual allocation of attention (i.e., information about when and where a person is attending) during navigation, improving the breadth of instrumentation in this area. Use of VARFA to instrument vision could be used to better inform fall risk assessment by providing behaviour and context data to complement instrumented e.g., IMU data during gait tasks. That may have notable (e.g., personalized) rehabilitation implications across a wide range of clinical cohorts where poor gait and increased fall risk are common.

**Supplementary Information:**

The online version contains supplementary material available at 10.1186/s12984-024-01400-2.

## Introduction

Falls can lead to loss of independence and even death [1, 2]. Identifying those at risk of falling is an important clinical task often conducted in e.g., those with visual impairment [3], and the elderly [4–6]. Equally, fall risk assessment is of notable importance and pragmatically useful in people with a movement disorder, such as Parkinson’s disease (PD) [7–9] or Stroke [10–13] due to observable functional deficits in motor control. Additionally, assessing fall risk is equally important during pregnancy [14] where a third of pregnant women may fall [15]. In fact, there is a significant increase in falls from pre-pregnancy to the 3rd trimester which cannot be fully explained by morphological [16] or biomechanical [17] changes.

A comprehensive fall risk assessment is multifactorial and a time-consuming process including but not limited to medication review, cognitive screening, detailing a history of falls, as well as evaluating gait, balance [18], and environmental hazards or hazardous activities that have been documented in some cases to be responsible for 50% of falls [19]. For timeliness in many settings, assessing gait alone is usually conducted to evaluate intrinsic fall risk [20]. That is convenient as gait is a good marker of global health [21] and fundamental to many activities of daily life [1]. Consequently, a gait assessment with positive outcomes from subjective evaluation (by an assessor) provides insight into the patient’s independence and ability to ambulate with minimal fall risk. As described, an assessment is typically conducted by manual observation alone, where an assessor examines a person’s gait during a scripted task (i.e., walking protocol). Often, a protocol may include navigating (walking around or over) obstacles [22–25], deliberately challenging the person by increasing gait demands [26]. Yet, that also places extra burden on the assessor, challenging them to carefully observe the person’s gait during a more complex task. Instrumentation is needed to optimize assessment protocols while providing high resolution objective fall risk data.

The integration of digital technology as an objective standard in fall risk is not routine. While digital tools may provide clinicians with high-resolution data to potentially aid in determining a patient’s fall risk, there is still ongoing work to be done in understanding their full utility and developing appropriate methods. In recent years, technology has matured to include a wide selection of digital tools. Of course, 3D motion capture systems are a perceived gold/reference standard for human movement analysis, but it lacks practicality and deployment in habitual settings. Moreover, reflective markers require timely application. In contrast, wearable devices (i.e., inertial measurement units, IMUs) are quickly attached and provide clinically relevant gait characteristics to a millisecond resolution in any environment [27–30].

An objective gait assessment to inform fall risk is usually conducted within a laboratory with a single IMU on the lower back [30]. Typically, participants are then asked to undertake a protocol representing walking challenges in daily life [31, 32], like obstacle crossing [25]. However, a key IMU limitation is the provision of inertial gait data only without any insights into navigating behavior and visual attention allocation to environmental/extrinsic details. Accordingly, there is no absolute clarity to understand how gait and fall risk is influenced by other intrinsic (e.g., visual attention) or extrinsic (e.g., obstacles) factors. For example, a comprehensive instrumented assessment would better understand how those being assessed allocate visual attention along their walking path for safe navigation while also determining the role of attention when e.g., peripheral obstacles cause a distraction. Supplementing IMU data with video data from video-based eye tracking wearable glasses could better define intrinsic and extrinsic factors, providing a contemporary and pragmatic approach to fall risk assessment with easily attached wearables. (Indeed, eye tracking offers an avenue for exploring neurocognitive changes as a reason for increased falls incidence.)

Commercial eye tracking glasses capture high quality video data and often in the standardized MP4 format with a resolution of 1920 × 1080. The video contains a superimposed crosshair to display eye location. Accordingly, videos contain data on the general environment and specific objects of where the wearer is looking but data processing of eye-tracker videos is extremely time consuming and needs to be automated to allow clinical application [33]. Including eye tracking (to identify an object/obstacles of interest) with IMU data during a range of simulated free-living tasks (e.g., obstacle crossing) would provide a novel approach for simultaneously instrumenting visual attention during gait within a fall risk assessment. To accomplish this, a suitable methodology to instrument visual attention from video data must first be established as none currently exists. Accordingly, a novel vision-aided fall risk assessment (*VAFRA*) is proposed in this study.

### Instrumenting vision: a computer vision approach

Video-based eye trackers can help understand the allocation of visual attentional (and visual function) [34, 35]. Attention relates to the ability to focus on a task and within the context of gait assessment for fall risk could relate to when and how long one fixates on a hazardous obstacle [36]. It has been shown that duration of fixation and therefore attention on an obstacle is linked to avoidance or clearance and risk of tripping [37]. Quantifying fixation time on an obstacle can reveal the relationship between attention, obstacle avoidance or clearance, and fall risk. During navigation, the visual system provides critical information about factors like object depth, one’s heading direction, and time to contact with an object, but it also includes visual acuity, contrast sensitivity, depth perception, and visual field integrity, among others [38]. Those aspects of visual function contribute to detecting, and perceiving extrinsic/environmental cues, including obstacles, during walks.

Eye trackers can help assess attention (and visual function) by quantifying gaze patterns, saccades, and fixations during walks with obstacle avoidance or crossing tasks [39]. Those with compromised visual function may exhibit inadequate gaze patterns [40] that can lead to inadequate attention allocation to obstacles which, in turn, can increase the risk of tripping or falling [41]. Conversely, those with intact visual function are more likely to allocate appropriate attention to obstacles and hence successfully traverse around them, reducing fall risk. Despite the promising opportunities with eye-trackers, there are two key challenges to be overcome: (i) the time-consuming review of video data with manual labelling of frames [33, 42] and (ii) objective identification of where the person is looking, and hence attending. The latter is complicated by any extrinsic obstacle which may impact walking and/or other items in the environment. Often, labs strive to maintain a clean environment by removing non-critical equipment but often that is not practical [33]. This is especially true in ecologically valid situations (i.e., everyday life), where distractions are common and can significantly contribute to fall risk. Moreover, many settings where fall risk assessment occurs (e.g., inpatient setting) have similar challenges to minimize distractions while maintaining optimal conditions.

An automated approach is necessary to analyze attention and visual function using video eye-tracking within the context of fall risk assessment, complementing the developments in IMU data processing for fall risk evaluation [30]. In fact, many populations do not exhibit their true motor deficits until attention is divided [43, 44] and other real-world motor tasks like object avoidance can be best measured through the person’s visual recognition [45]. Better (instrumented) methods are needed to measure visual attention within real-world motor tasks. Artificial intelligence (AI) methodologies within computer vision (CV) enable automated instrumental approaches with e.g., object detection. CV algorithms label many video frames in a timely manner, classifying environmental contexts while informing attentional behaviors from eye tracking i.e., automating eye location overlapping with extrinsic factors like obstacles, distractions and/or hazards. Importantly there are attainable contemporary CV methodologies that are state of the art and can be tailored to each specific use case e.g., YoloV8 [46].

To date, instrumented gait has received extensive research focus but fall risk assessment through gait alone, although useful, remains limited. Here we propose a deep learning-based object detection algorithm (*VAFRA*) for the novel instrumentation of allocation of visual attention (gaze) and contextualization of video data to better inform fall risk assessment within a lab during an obstacle crossing based continuous walk. The work is important as it presents a novel approach to better inform lab-based assessment of objective fall risk to advance approaches to rehabilitation via contemporary technologies. Specifically, we suggest pragmatic models to help instrument visual attention components during walks to better inform how video and eye-tracking can be used during fall risk assessments. The technology employed can capture and analyze gaze patterns and environmental interactions in a manner that is not dependent on the visual acuity of the user.

The paper is structured as follows. In the Methods and Materials section, we provide an overview of the participants recruited, lab-based protocol and discussion of the dataset to train a model. That section also details (i) the mechanics detecting eye location and overlap between objects as well as determining object row and (iii) a U-Net approach for creating segmentation masks for the walking tracks/paths. The Results section provides preliminary statistics comparing foot clearance during attention vs. distraction as defined by the model. The Discussion provides insights to the instrumented approach with limitations but highlights future application to assess fall risk in cohorts needing this proposed approach.

## Materials and methods

### Participants

This research was approved for human subjects’ study by the Washington State University institutional (ethics) review board (IRB# 17442). All participants provided written informed consent. The study recruited a total of twenty healthy pregnant women (29.9 years old ± 4.9 years, 66.0 kg ± 10.5 kg, 166.0 cm ± 6.7 cm). All female participants were in approximately their 13th week of gestation (± 1 week). Recruitment was conducted through flyers distributed during their initial obstetrician visit. They volunteered by calling into an enrollment person/researcher for screening. They were excluded from participating if they were considered a high-risk pregnancy, unable to walk unassisted, had a cognitive inability to read and understand instructions, or if they could not commit to longitudinal testing for the entirety of the pregnancy.

### Protocol

**Fig. 1:**
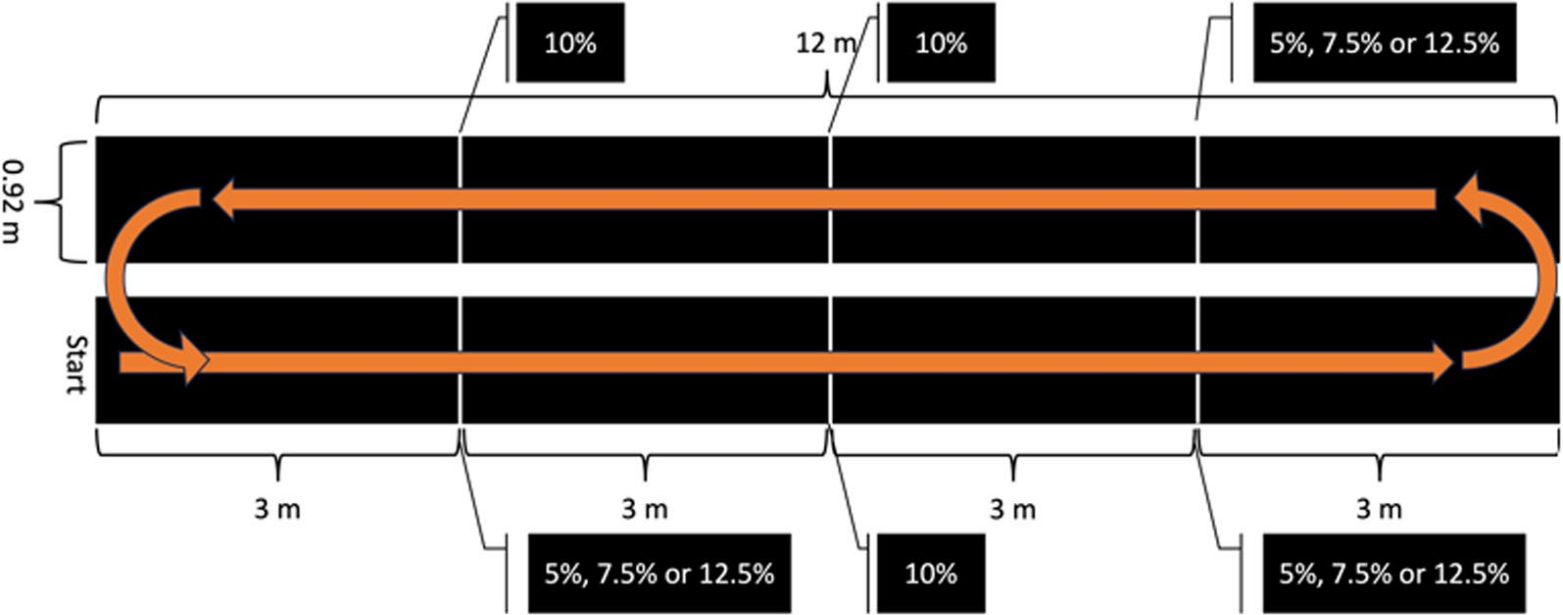
The walking path route undertaken by all participants during testing. Upon starting, participants were tasked with crossing obstacles at 3 m intervals. Obstacle heights were set at a percentage (%) of the participant heigh during each walking trial

Participants were enrolled as part of a larger follow-up (longitudinal) study examining fall risk in pregnancy. Each follow-up timepoint (n = 5) contained a wide testing battery and each lasted approx. 60-min. Accordingly, about 100 h of video data were accumulated. During each testing session, participants wore eye tracking glasses (Tobii Pro2, Stockholm https://www.tobii.com), which captured environment/lab video data at 24 frames per second (fps) from test start to finish along with the participants gaze at each frame (1920 × 1080 px). The lab comprised two walking tracks/paths within a continuous loop with intentionally placed hurdles and distractors, Fig. 1. Six fully visible white PVC pipe obstacle hurdles were placed 3 m apart along the 12 m long, 0.92 m wide, two-sided black walking path. Some hurdles were always set to 10% body height, while surrounding hurdles were randomly assigned to 5%, 7.5%, 10%, and 12.5% of body height of the tested participant.

### Dataset

Video data spanning the full 60-min per participant were utilized to train the proposed model. This was due to the frequent examples of more obscure angles and head positions captured when the participant was not performing a direct 2-min walk test and may for example be standing and talking to a researcher between tasks while looking around the lab setting. That aided the model to generalize to more diverse scenarios like rare head angles during the test. A key advantage to the data being captured within a controlled setting is homogeneity. Specifically, within all videos captured from participants, variables such as lighting, hurdles and video quality remain similar. For use within the produced dataset the frames of the captured footage were extracted and labelled using a Python-based tool [47] with example classes being: hurdle, tennis ball, animate distractor, bucket.

The labelling process resulted in a dataset consisting of 987 labelled frames and 18 classes across all frames. These classes represent a variety of objects or obstacles that are pertinent to determining whether a participant is paying attention (i.e., to obstacles along the path) or distracted. Of these 18 classes 3 are defined as “core objects” being tennis ball, support, and hurdles as these are the direct objects and points of investigation for the task. The labelled information was extracted using the inbuilt functionality of the label producing software. The images folder contained the full resolution raw images with accompanying labelled information stored in the annotations folder in*.txt* format. Annotations contained a line for each object detected within the scene holding the object class id and object bounding box coordinates within the *x* mid, *y* mid, width, height format.

### Object detection model (ODM)

**Fig. 2:**
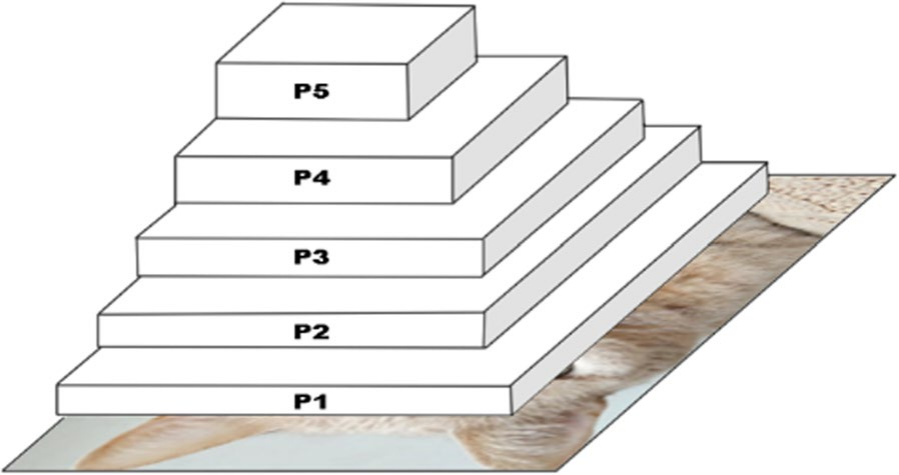
Example backbone architecture of the YoloV8 feature extraction model

Model implementation was performed using the Python-based deep learning library *PyTorch* and the *Ultralytics* suite of available Yolo algorithms. The final object detection algorithm used was the latest YoloV8 network [46]. That version was chosen as it has been shown to have more accurate results on images and video within 1.3 ms speed per image size at 640 × 640 (to be used in this study) compared to YoloV7 [48]. This architecture (Fig. 2) takes the image as input and feeds it through a series of convolution, pooling and batch norm layers before outputting predicted classes and bounding box coordinates on the extracted features.

The output from the model was then further enhanced using non maximum suppression, used to remove duplicated bounding boxes and reduce noise in detection based on intersection over union (IoU) metrics (i.e., overlap between predicted bounding boxes and ground truth annotations). Given the minute pixel data required to accurately classify important obstacles within the track, the model was trained on images resized to 640 × 640 px to retain ample image information while balancing performance. The requirement was further aided using distributed focal loss (DFL) which is a custom loss function used for improving the ability of models to identify small objects within images (Eqs. 1–4), which was a core requirement for the dataset and to also aid with class imbalances within the training data.

1 $$s_{j} = \frac{1}{{N_{j} }}\mathop \sum \limits_{i} \left[ {t_{i,j} \times \sqrt {\frac{{w_{j} }}{{h_{i, j} }}} } \right]$$

2 $$w_{j} = \frac{s}{{h_{j} }} + \epsilon$$

3 $$focal_{l} oss_{i,j} = focal_{l} oss_{i} \times \sqrt {\frac{{w_{j} }}{{h_{i, j} }}}$$

4 $$DFL\left( {p, y} \right) = - \alpha \left( {\frac{{n_{y} }}{n}} \right)(1 - p)^{\gamma } \log p$$

Equations: DFL Loss equation, where s is the average object size for the batch, N_j_ is the number of anchor boxes in the batch with ground-truth label h_j_ [2] is the height of the ground-truth bounding box for anchor box i with label j, epsilon is a small constant to avoid division by zero, and focal_loss_[48] is the focal loss for anchor box i with label j. The DFL loss is computed for each class j separately, and the final DFL loss is the sum of the DFL losses for all classes:

The training process was conducted within a Windows based Python 3.8 environment, on a system containing an RTX 3070 graphics card, Ryzen 7 3700X CPU and 24 GB of RAM and took ~ 3 h to train over 100 epochs. The dataset was split using a pragmatic 80:20 train-test ratio outputting evaluation metrics across both training and validation examples: *train/box_loss*, *train/cls_loss*, *train/dfl_loss*, precision, recall, mean average precision (mAP50 and mAP50-95), *val/box_loss*, *val/cls_loss* and *val/dfl_loss*.

### ODM: eye location

**Fig. 3:**
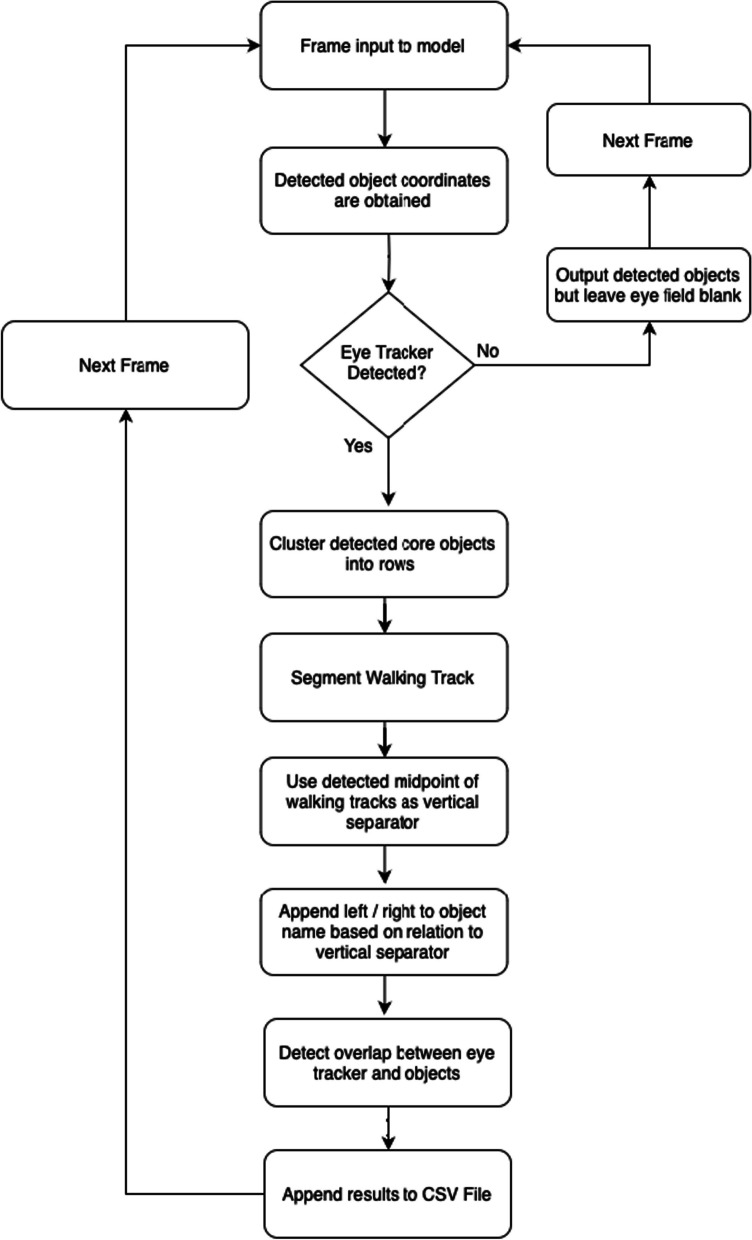
Flow diagram illustrating the deployment of the proposed AI model and its accompanying mechanics throughout a video

Classification of the objects provided context to the video data but when considered in isolation, provided little meaningful information. To automate the detection of where visual attention is, a mechanism is required to provide information (Fig. 3). Within the model an algorithm was implemented to detect overlaps between the bounding box coordinates using the × *1*, *y1*, × *2*, *y2* format. *Algorithm 1* outlines the process, by performing a *for* loop over each detected object, the coordinates are input to the overlap detection function taking the coordinates as arguments. Those coordinates are then compared with the stored eye tracker coordinates returning *true* if an overlap is detected.

### ODM: object row mechanic

Whilst the proposed lab setup mimics that of an optimal walking path for gait assessment [25], it also provides the inclusion of potential distractors and hurdles along the path. The spatial context of these distractors and hurdles are vitally important for inclusion within the model given their clinical significance and implications for assessing a participant’s visuospatial attention and ability to navigate environmental obstacles. To achieve this, detection of what the participant is looking at is performed first, followed by the classification of what row the object belongs to, appending this provided context within the CSV file (e.g., tennis ball row 2) upon completion.

**Algorithm 1:**
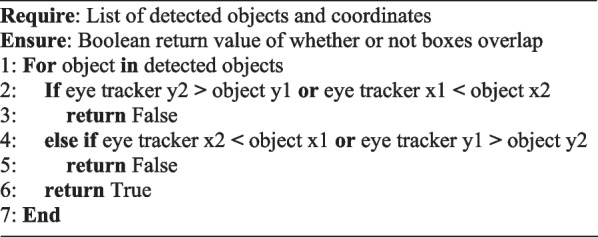
Algorithm for detecting bounding-box overlap

**Fig. 4:**
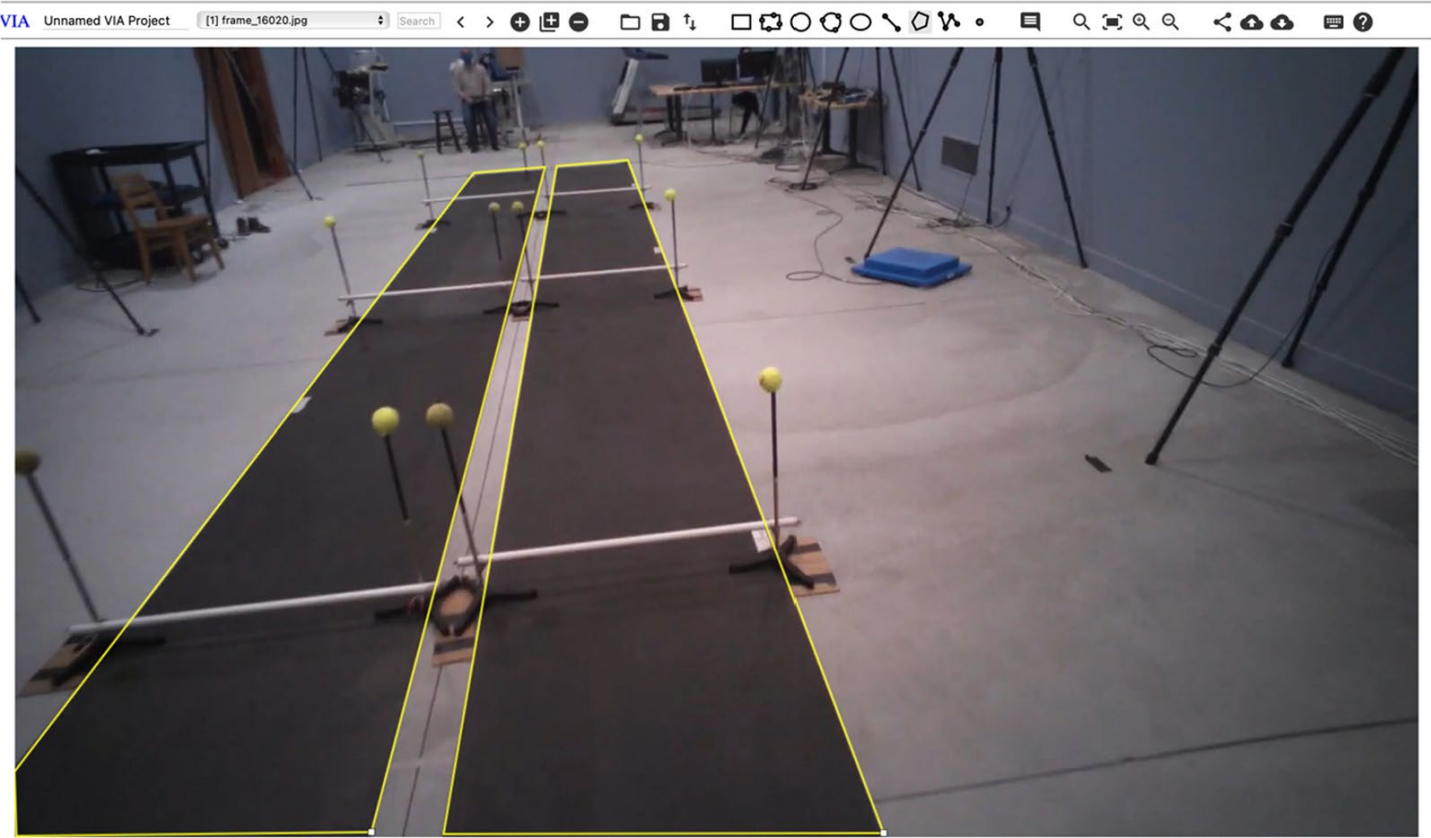
VGG image annotation tool, used to create the segmentation masks

When navigating the hurdles, a participant will encounter up to three sequential hurdles along each track/path, and it is important to understand which row the participants attention is on. For example, if it is known that the participant is looking at the immediate hurdle, it can be inferred they are paying attention to the hurdle and planning safe crossing (no contact). This assumption is based on typical gaze behavior observed in most individuals. However, we acknowledge that there may be exceptions, particularly among experienced participants or those familiar with the path. It can also be inferred that if the participant is not paying attention to the nearest hurdle before crossing, they are distracted. Here, across all scenes involving obstacle crossing, the same core objects are present and organized along the walking path into rows (Fig. 4), (i) a set of tennis balls (at each side of the walking track and used by the participant to judge horizontal opening size, defined as the horizontal distance between the two balls;), (ii) supports (are used to hold up the tennis balls and can also be used by the participant to judge opening size) and (iii) a hurdle (obstacle to be navigated by participant).

Given the consistent spatial relationship of these objects the vertical pixel coordinates can be used to begin to cluster these objects into their respective rows *Algorithm 2*. The algorithm first sorts the detected tennis ball objects based on their Y positions. Then, it iterates through the sorted list, calculating the distance between each consecutive pair of balls. If the distance is less than 50 pixels, the balls are considered to belong to the same row and are added to the current row array. If the distance exceeds 50 pixels, the current row is appended to the rows array, and a new current row is initiated to begin capturing the next set of balls within the same row.

**Algorithm 2:**
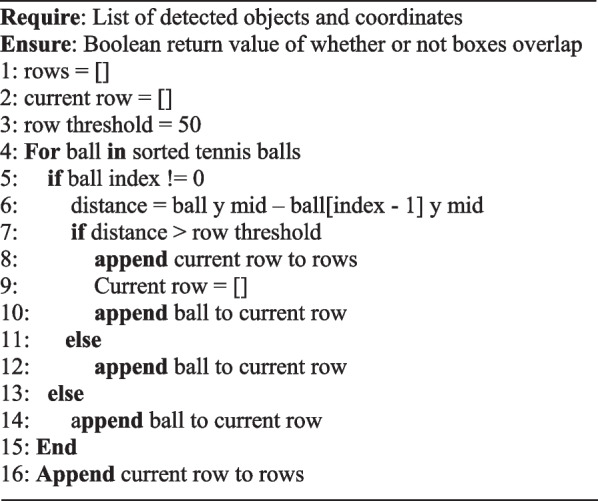
Algorithm for clustering balls

Once the algorithm for sorting hurdles into clustered rows was established, the loop responsible for classifying the actual row of the objects was created. *Algorithm 3* gives each detected object an associated row by looping over every detected object and determining the object’s midpoint. With this information deduced and each ball clustered into its respective row, the *y* point of the object can then be compared with the detected row lines. Whichever row is determined to have the least absolute difference is classified to be the row of the object.

**Algorithm 3:**
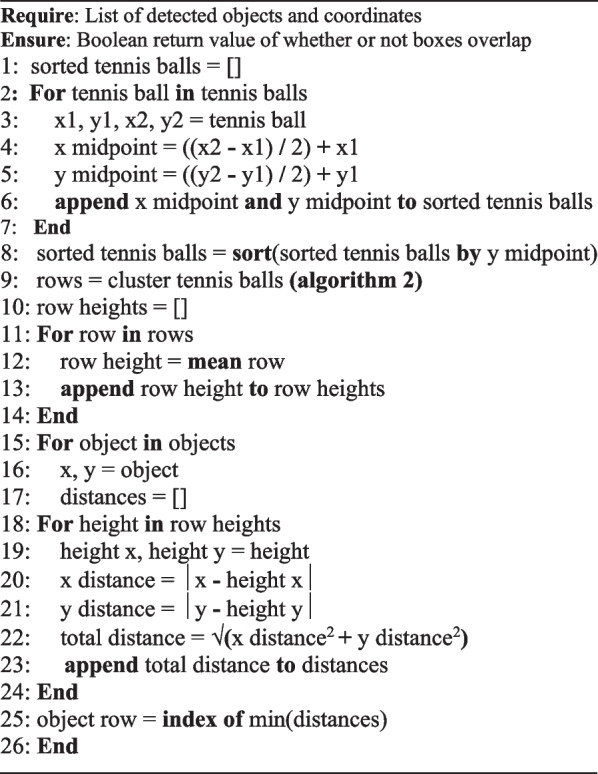
Algorithm for assessing object row

### Track segmentation model (TSM)

If participants are looking downwards at the track/path ahead of their immediate foot placement, this can provide context i.e., thinking about foot placement. Determining the exact spatial location of the participant’s walking path is more difficult, because a more detailed classification is required compared to general object detection. To address this, a further segmentation model was developed and deployed to provide a pixel-wise segmentation mask for exact track location. This means that the exact location of the tracks themselves were detected not just a general bounding box. To develop this model, the same process for dataset collection was utilized as with the object detection tool. The videos were broken down into component frames to be used as images within the dataset. To create the segmentation masks (black and white images containing white pixels only where the regions we want the AI to detect are) the VGG image annotation tool [49] was used (https://www.robots.ox.ac.uk/~vgg/software/via/) Fig. 4.

**Fig. 5:**
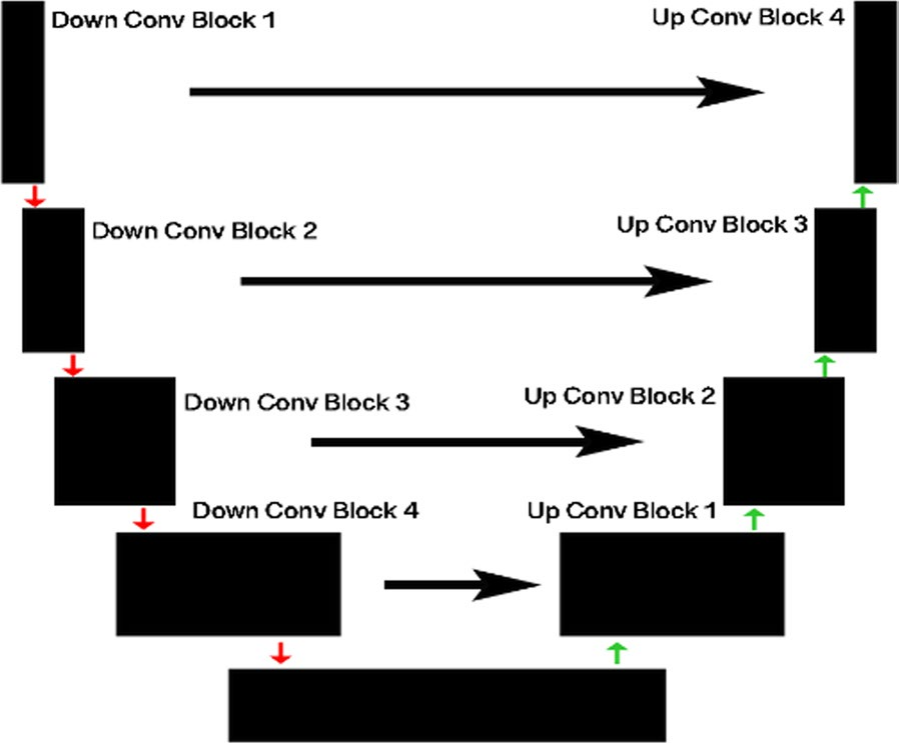
Visualization of the U-Net architecture that depicts how an image passes through the network

Using VGG, the tool for creating segmentation masks to be used in AI models, a dataset of 388 frames and accompanying binary segmentation masks were created. This dataset was then used to create a U-Net based segmentation network (Fig. 5) with *PyTorch*. This model was then trained within the same Python 3.8 environment using a Ryzen 3700x, 24 GB of RAM and an RTX 3070ti based machine over a course of 100 epochs. After gaining a binary (white/black) segmentation mask of track location, detection of overlap between the eye location and track mask (black and white segmentation masks where only the location of the tracks are white) can be identified, Algorithm 4.

**Algorithm 4:**
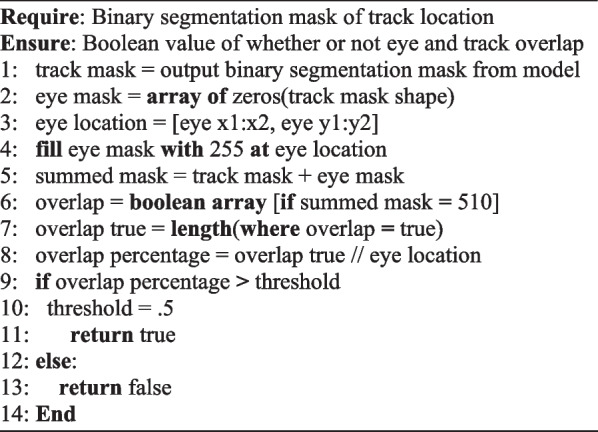
Algorithm for detecting track overlap

### TSM: left/right object direction

With a methodology in place to assess an object’s row, a methodology for detecting which track a set of objects belong to is required (left or right). Understanding which side of the tracks an object belongs to is an important classification to assessing whether or not the participant is distracted (like paying attention to your driving lane vs oncoming traffic on a two-lane road). The track being actively navigated will always be on the right from the participants perspective, meaning any attention paid to objects on the right track will be relevant to navigation planning either immediately or in the near future. Conversely, attention paid to obstacles on the left track indicates a distraction, as when they are visible they will be beyond the immediate area of the participant. A further algorithm (*Algorithm 5*) can be implemented to attain what side an object is on relative to the participant by inferring the mid-point between the different segmented track points.

**Algorithm 5:**
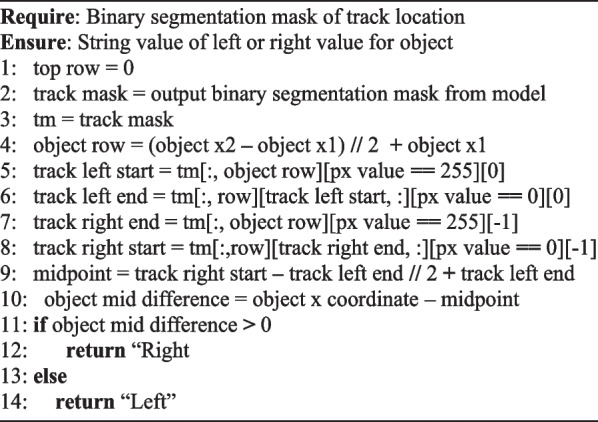
Algorithm for left/right side detection

## Results

### Dataset

Currently the main object detection dataset contains 958 fully labelled and annotated frames covering various aspects of the 2-min walk. For the track detection segmentation model a dataset of 358 frames and accompanying binary segmentation masks were collected and finalized for training the models. The dataset for both models cover all aspects of different lighting conditions and laboratory layouts.

### Object detection model (ODM)

**Fig. 6:**
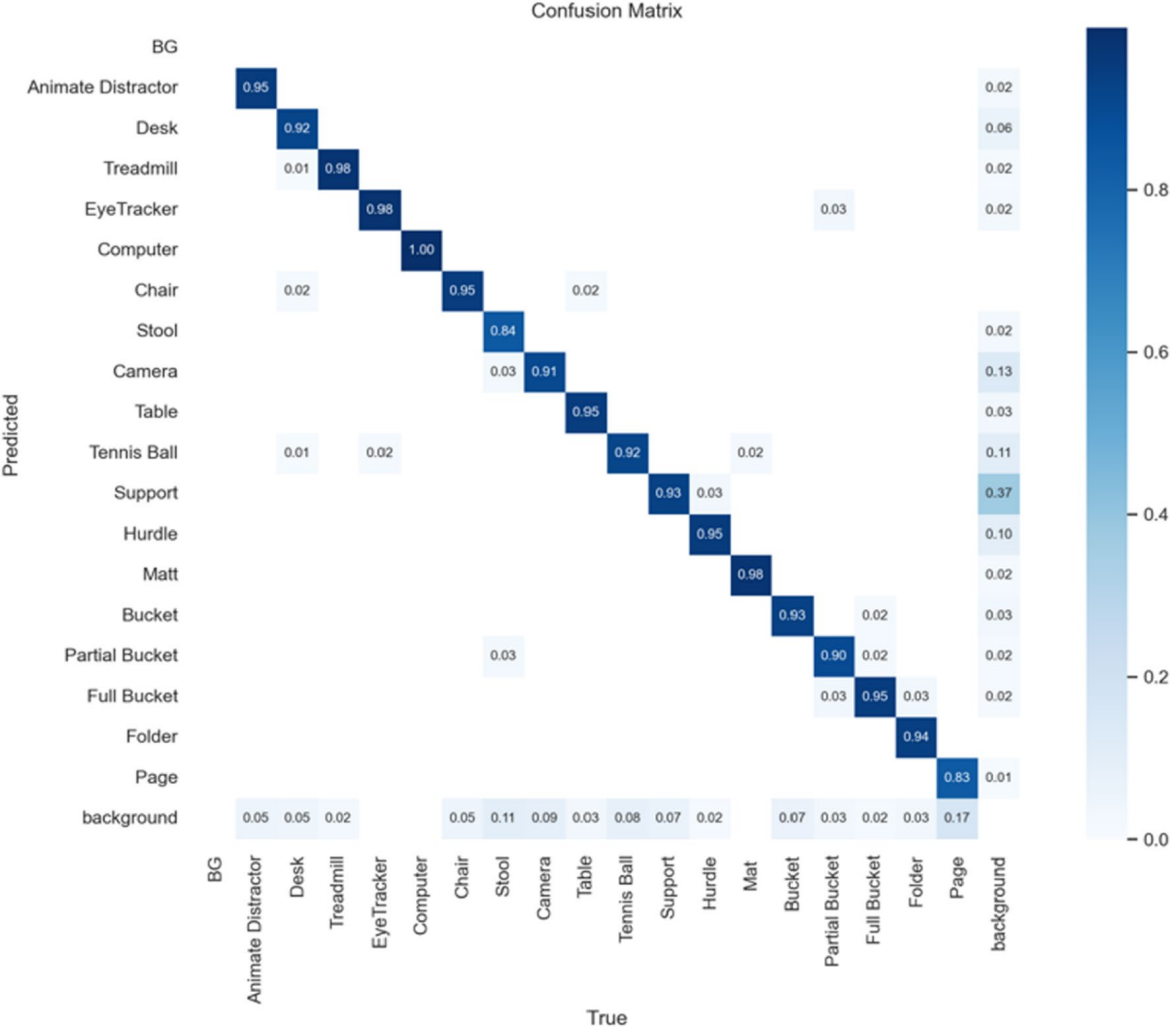
YoloV8 model confusion matrix when tested on validation dataset

A range of different output evaluation metrics were obtained from the model training (see supplementary materials, Fig. S1). To select the best model, only metrics obtained on the validation dataset were considered, with the mAP50 (mean average precision at 0.50 intersection over union threshold) used as the metric for determining the best performing model. The mAP50 being an accuracy metric that measures the percentage of predictions in object detection that have at least a 50% overlap with the ground truth annotations. The model peaked in performance at epoch 64 with an mAP50 of 0.93 (Table 1) indicating excellent model performance. This was further validated by the results from the confusion matrix (Fig. 6) calculated over the validation dataset showing excellent performance across all classes (> 0.80). Initial viewing of the confusion matrix showing results from the validation set may seem to indicate overfitting or data leakage. However, the high values for some classes (e.g., Computer = 1.0) appear to be caused by strong class imbalance with very few examples being included within the validation set due to the small number of frames in general containing this object. To mitigate the effects of the class imbalance the model was trained using the DFL loss function by applying weightings to samples based on more hard, misclassified examples during training.

**Table 1:** Yolov8 object detection model validation metrics

Epoch	Precision	Recall	mAP50	Box loss	Cls loss	DFL loss
62	0.89	0.92	0.92	1.06	0.58	1.02
63	0.88	0.93	0.92	1.06	0.57	1.03
**64**	**0.90**	**0.93**	**0.93**	**1.06**	**0.57**	**1.03**
65	0.89	0.92	0.92	1.07	0.57	1.03
66	0.88	0.91	0.92	1.06	0.57	1.02

Typical for best epoch indicated in bold

*DFL loss* Distribution focal loss, *Cls loss* Class loss, *Box loss* Bounding box loss

### Track segmentation model (TSM)

Findings indicate that the segmentation model achieved a mean IoU score of 0.82 (Table 2), signifying a significant degree of overlap between the predicted segmentation masks and the ground truth labels on the validation data indicating its ability to generalize well to unseen data. The model’s convergence at epoch 6 implies that the model training process was effective and efficient. These results demonstrate the potential of segmentation models to effectively detect walking tracks in lab-based images, which has significant implications for gait analysis in research studies. Future research will focus on implementing this model to larger datasets and refining its performance to improve its accuracy and robustness.

**Table 2:** Segmentation model convergence at epoch 6

Epoch	Val IoU	Val loss
1	0.12	59.23
**6**	**0.82**	**36.33**
12	0.78	37.53

Typical for best epoch indicated in bold

## Discussion

This paper proposes a vision-aided fall risk assessment (*VAFRA*) algorithm for automating the processing and labeling of contextual extrinsic (environmental) information obtained from head-mounted wearable eye tracking glasses/devices. To the authors' knowledge, no other research has proposed this method to better inform fall risk assessment, complimenting instrumented approaches as seen with IMU-based gait. Accordingly, authors believe this is the first study to propose object detection algorithms for contextualizing wearable eye tracking data within a lab-based environment for fall risk assessment. Whilst other studies have proposed the fusion of AI, wearable eye tracking glasses and kinematic data [50] this study presented here is the first to utilize the visual attention (gaze) function from the glasses to investigate the visual attentional of a participants navigation.

The model utilizes state-of-the-art object detection algorithms (YoloV8) trained on a unique laboratory-based dataset. Yolo was chosen given the core requirement of the system to be able to contextualize the environmental objects in a time efficient manner for use in wider studies. At present whilst other state-of-the-art object detection algorithms exist [51–54], they do not offer the speed required of such applications that is currently available using the yolo series of algorithms as shown in their use in other cases [55–58]. Our model demonstrates accurate classification and localization of objects and hazards within the lab environment, as well as accurately determining the participant's gaze location. By providing object coordinates and eye fixation location, a simple box overlap algorithm (*algorithm 1*) offers a robust and generalizable approach for automatically detecting eye gaze direction to infer where a participant is attending in space. The more challenging aspect of utilizing the box overlap algorithm involved the quantification of what row and side an object was relative to the observer. To quantify the row of the object, an algorithm for clustering detected objects into respective rows based on Y coordinates was implemented. However, that same approach could not be performed to quantify whether the object was on the left or right of the observer. To quantify left and right, an additional segmentation algorithm was created to accurately detect walking tracks. Given the layout of the walking tracks, a midpoint was clearly visible between the two tracks. That midpoint, when detected through the gap in the segmentation masks, accurately separated objects by providing an angled vertical line separator for classifying the objects side. Importantly, that did add to the computational complexity and performance overhead of the full system. Evaluation of the model's performance yielded promising results, showing significant accuracy across all objects (83%–98%). Overall accuracy of detected objects was demonstrated by a mean average precision at 50% intersection over union (mAP50) value of 0.93 (Table 1) on the validation dataset. The robustness of the proposed model was further confirmed by the values depicted in the confusion matrix (Fig. 4), with most classes achieving a score > 0.90. Interestingly, classes with limited training examples achieved respectable scores of > 0.83. Whilst the model achieved high accuracies across all classes some causes for concern are also present. A low number of samples within a data set may lead to inaccurate results, principally the mAP50 score of 1.0 for the computer class. Upon investigation it was seen that the computer class had an incredibly limited number of samples within the dataset with even fewer belonging to the validation dataset (results in Fig. 6). The combination of very few validation examples and use of fine tuning on the Yolo algorithm of which its original training data included many examples of such a class would give reason to believe this was the cause of such a high score rather than overfitting or data leakage.

Notably, the model achieved an impressive 0.98 detection accuracy for eye-tracker location. This indicates the model's ability to accurately determine the participant's gaze location, providing an effective means of understanding their current focus of attention. The proposed model represents the first steps toward an AI-based context driven breakdown of free-living fall risk assessment [28]. Indeed, adoption of a comprehensive approach may help overcome ethical challenges with video capture in the wild [59]. VAFRA uniquely applies deep learning techniques to process video data from eye-tracking glasses, which can capture the participant’s gaze location and attention allocation. Existing methods for fall risk assessment typically focus on either single sensor based inertial measurements [27, 60] or providing environmental context without the use of eye tracking technology, neglecting the attentional aspects of fall risk [28]. In time, models like VAFRA could be deployed within fall prevention strategies like in [61], except with mobile based cameras more reflective of real world scenarios. Object detection algorithms have been examined for those with visual impairment. One example [62] utilizes a multimodal approach consisting of a walking aid mounted mobile camera and depth sensor. However, the object detection algorithm is not categorically described, as the cited work uses TensorFlow application programming interface (API) with the MS COCO dataset [63]. Although the referenced paper does not provide comprehensive evaluation metrics and use of a publicly available dataset only it helps to showcase the use of object detection within egocentric contextualization. In contrast, Joshi et al. [55] created an on board visual impairment aid that used a custom primary capture dataset and the YoloV3 algorithm for object detection. However, that work also fails to provide robust validation metrics by describing objects as correctly recognized at an average of 95%, when compared with the more stringent and robust metric of an mAP50 value evaluated on our model where 0.93 suggests that despite its more homogenous dataset has an incredibly strong accuracy for wider application. For example, Sankarnarayanan et al. [64] use a publicly available image dataset (Google Open Images v6) to show how improvement in mAP50 value from 0.31 to 0.50 is described as moderate performance. Although the dataset utilized by Sankarnarayanan et al. is less homogenous (91,167 images, 20 classes) across a range of environments compared to our novel dataset (958 images, 19 classes) in a single environment it serves as a benchmark for detailing how effective VAFRA is (0.93 mAP50) on a small training dataset.

### Informing gait rehabilitation

One of the primary benefits of this system is its capacity to automate the analysis of extensive datasets. With over 100 h of footage to review within our use case, traditional manual analysis would be impractical and resource intensive. The developed model's ability to detect distractions and obstacles, coupled with determining whether these elements overlap with the participant’s gaze, significantly reduces the time and labour required for comprehensive data analysis. This system now enables an efficient methodology for determining correlations between, for example foot clearance height between those participants paying attention to an upcoming obstacles vs those that are not or extraction of what objects are causing distractions on larger scale datasets gathered within the lab. Although not the focus here, a further interesting use of *VAFRA* would include the examination of how participants may change their strategy to the gait tasks over different time points. Specifically, examining the intrinsic response (i.e., visual attention and gait) due to (repeated) exposure to distractors within the environment. Understanding these dynamics can inform clinical practices, leading to the development of targeted interventions and therapies aimed at improving gait and balance in this population. The system’s adaptability extends beyond pregnant women, offering applications in various fields such as sports science, ergonomics, and neurological rehabilitation, where the interaction between attention and movement is critical. The precision of the object detection model ensures high accuracy in identifying distractions and obstacles, which is essential for drawing reliable correlations between visual attention and kinematic responses.

### Strengths and limitations

One important limitation of this research is that the findings may not be generalizable to other labs or indeed non-lab-based conditions. That is because the object detection model was developed and evaluated in a controlled lab environment with data from a single location. Although a large volume of video data was used the number of participants with eye-tracking and gait data was conservative.

### Future work

Future work will be needed to assess the generalizability of this approach to more complex, real-world environments. Specifically, work from this study will aim to deploy this form of technology within free-living environments to assess other cohorts with elevated fall risk, such as people with Parkinson’s Disease (PD). For example, using a model like *VAFRA* with video-based eye tracking glasses during IMU-gait assessment could lead to a comprehensive understanding of real-world visual cues to mediate PD gait and reduce falls [65]. Deployment beyond the lab/clinic will improve our understanding of gait patterns in real-world settings and help to develop personalized interventions to improve mobility and prevent falls in several clinical populations that have increased risk.

One of the major challenges for the model concerns its generalizability to the range of different lighting conditions and range of potential objects–both of which are highly variable in real-world settings. Regardless, overcoming that challenge is necessary to advance VARFA and instrument vision more broadly to compliment the advances in instrumented gait and to better understand free living fall risk in a range of clinical cohorts. One topic of work that should be considered as part of refining instrumenting vision beyond on the lab is the examination of other CV algorithms (e.g., other Yolo versions) to assess their performance and proficiency.

## Conclusions

The proposed VAFRA methodology helps instrument eye gaze associated with visual attention during walks to better inform behaviour and context, complimenting gait characteristics in a more holistic fall risk assessment. It supports the use of object detection models in complex lab environments, where AI-based contextual information is important for understanding gait patterns and detecting abnormalities. Use of object detection models can help instrument vision from video-based eye-tracking glasses within lab-based fall risk assessment and results demonstrate potential to accurately contextualize gait data. Instrumentation beyond the lab is the next step for habitual fall risk assessment.

### Supplementary Information

Supplementary Material 1.

## Data Availability

The data that support the findings of this study are not openly available due to reasons of sensitivity and are available from the corresponding author upon reasonable request.
