# Supplementary material for: Enhancing fall risk assessment: instrumenting vision with deep learning during walks
Source: J Neuroeng Rehabil. 2024 Jun 22;21:106. doi: 10.1186/s12984-024-01400-2 (PMC11193231; doi:10.1186/s12984-024-01400-2)
Supplement: Supplementary file 1 — Supplementary Material 1. [file 12984_2024_1400_MOESM1_ESM.docx]

**Supplementary Materials**

This supplementary file contains additional graphs related to the training metrics used in our study. Due to space constraints in the main paper, these supplementary materials are provided to offer a comprehensive view of our experimental results. The included graphs complement the analysis presented in the main text and provide further insights into the performance and distribution of data in our study. We encourage readers to refer to these supplementary materials for a more detailed understanding of the experimental findings.


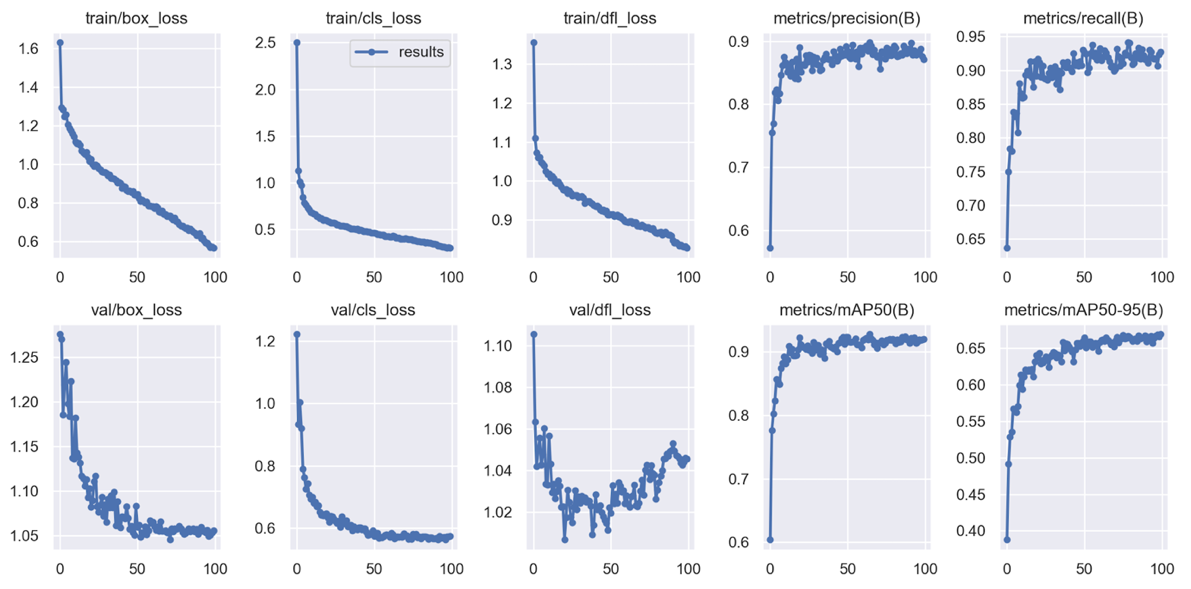


**Fig. S1.** Range of full output metrics from the YoloV8 model over the 100 epochs.
